# Natural product therapy in diabetic kidney disease: emerging multiomics-mediated signalling pathway and molecular target

**DOI:** 10.1186/s13020-026-01511-z

**Published:** 2026-09-23

**Authors:** Kai-En Wang, Qing-Qing Yu, Wen-Feng Wang, Yan-Ni Wang, Jie Yang, Hua Miao, Li-Min Liu, Ying-Yong Zhao

**Affiliations:** 1 School of Pharmaceutical Sciences, Zhejiang Chinese Medical University, No. 548 Binwen Road, Hangzhou, 310053 Zhejiang China; 2 Department of Clinical Research, Xianyang Hospital of Yan’an University, No. 38, Middle Section, Wenlin Road, Xianyang, 712000 Shaanxi China; 3 Department of Nephrology, The Second Affiliated Hospital of Zhejiang Chinese Medical University, Hangzhou, Zhejiang China; 4 State Key Laboratory of Kidney Diseases, First Medical Center of Chinese, PLA General Hospital, No. 28 Fuxing Road, Beijing, 100853 China

**Keywords:** Diabetic kidney disease, Natural products, Traditional Chinese medicine, Multiomics, Genomics, Transcriptomics, Metabolomics, Gut microbiota, Non-coding RNAs, AGE/RAGE pathway

## Abstract

Diabetic kidney disease (DKD) is one of the most common microvascular complications of diabetes, with high mortality and morbidity, and is a major cause of progression to end-stage renal disease (ESRD). Although inhibitors or blockers of renin-angiotensin system blockers have a beneficial effect on the treatment of DKD, most patients progress to ESRD and ultimately require renal replacement therapies. Extensive studies have suggested that natural products, as a mainly new drug source, has been demonstrated to be an important therapy for the prevention and treatment of DKD. Based on genomics, transcriptomics, proteomics and metabolomics technologies, the dysregulation of targeting multiomics-associated gut microbiota, non-coding RNAs, altered proteome and endogenous metabolites has recognized as a promising therapy for DKD. This review summarizes natural products, including traditional Chinese medicine formulas (Qitu Qushi formula, Jin Gui Ren Qi Pill and Huangkui capsules), single herbal extracts (*Salvia miltiorrhiza*, Korean red ginseng, *Abelmoschus manihot* flowers) and natural compounds (magnesium lithospermate B, thonningianin A germacrone, triptolide, hesperetin, astragaloside IV, icariin and kaempferol) as well as natural polysaccharides, protect against DKD by regulating novel multiomics-associated molecular mechanisms, including gut microbiota dysbiosis, aberrant non-coding RNAs transcription, and disorder of endogenous metabolites and associated with downstream diverse signals, such as IκB/NF-κB, JAK/STAT, MAPK and NLRP3-mediated inflammation pathways, AGEs/RAGE and Keap1/Nrf2-mediated oxidative stress pathways, PI3K/AKT/mTOR, AMPK and endoplasmic reticulum stress-mediated energy/autophagy pathways and TGF-β/Smad and Wnt/β-catenin-mediated fibrosis pathways. Therefore, the underlying molecular mechanism by which natural products ameliorate DKD by reshaping GM dysbiosis, restoring aberrant expression of ncRNAs and regulating metabolite disorder will provide new therapeutic targets for treatment of DKD of natural products. These findings expand our understanding of therapeutic effects of natural products on DKD and provide valuable information for clinical application of natural products. This review presents a concept-driven therapeutic strategy for DKD treatment and management.

## Introduction

Diabetes mellitus is the primary cause of chronic kidney disease (CKD) and renal failure worldwide [1–3], and diabetic kidney disease (DKD) is associated with excessive cardiovascular and all-cause mortality [4–6]. DKD causes from chronic exposure to hyperglycemia, resulting in progressive changes in renal structure and function [7–9]. DKD is characterized by increased glomerular and tubulointerstitial accumulation and deposition of extracellular matrix (ECM) components, such as type I and III collagen, fibronectin, α-smooth muscle actin, and vimentin [10–12]. Extensive studies have demonstrated that DKD is associated with a variety of molecular mechanisms, such as advanced glycation end products (AGE)/receptor for advanced glycation end products (RAGE), inhibitor of kappa B (IκB)/nuclear factor kappa B (NF-κB), Kelch-like ECH-associated protein 1 (Keap1)/nuclear factor erythroid 2-related factor 2 (Nrf2), transforming growth factor β (TGF-β)/suppressor of Mothers against decapentaplegic (Smad), Wnt/β-catenin, phosphoinositide 3-kinases(PI3K)/protein kinase B(AKT)/mammalian target of rapamycin (mTOR), Janus kinase(JAK)/signal transducers and activators of transcription (STAT), adenosine 5'-monophosphate-activated protein kinase (AMPK), autophagy, and endoplasmic reticulum (ER) stress signaling pathways [13–17].

Metabolic disorders, including obesity, hyperglycaemia, dyslipidaemia, ischaemia, and inflammation exacerbate renal function decline[18, 19]. Therefore, the key therapy to delay the progression of DKD remains blood sugar control and blood pressure decrease, with blood pressure was often decreased by using angiotensin-converting enzyme inhibitors and angiotensin type 1 receptor blockers [20–22]. Extensive research has highlighted glucagon-like peptide 1 receptor agonists, sodium-glucose cotransporter 2 inhibitors, and non-steroidal mineralocorticoid receptor antagonists can enhance renal function and reduce renal fibrosis in patients [23, 24]. Although these inhibitors and blockers showed the beneficial effects on the treatment of DKD, most patients progress to end-stage renal disease (ESRD) and ultimately require renal replacement therapies, such as hemodialysis, peritoneal dialysis, and transplantation [25–28]. However, these therapies are costly and not curative.

Natural products derived from natural sources, including abundant plants, microorganisms, and animals, show excellent efficacy for treatment and prevention of diverse diseases, with fewer side effects, synergy, and ability to reduce drug resistance [29–32]. Natural products, such as traditional Chinese medicines (TCM), serve as a major source of drug discovery and show great advantages in the prevention and treatment of kidney disease, including DKD [33–36]. With the development of emerging multiomics, such as metagenomics, transcriptomics, proteomics, and metabolomics, an increasing number of publications have highlighted that DKD is associated with dysbiosis of gut microbiota (GM), dysregulation of non-coding RNAs (ncRNAs), altered proteome, and disorder of endogenous metabolites [37–41]. Based on genomics, transcriptomics, proteomics and metabolomics technologies, the dysregulation of targeting multiomics-associated GM, ncRNAs, altered proteome, and endogenous metabolites has recognized as a promising therapy for CKD, including DKD [42–44]. This review explores potential alternative therapies of natural products for treatment of DKD by improving GM dysbiosis, aberrant ncRNA transcription, and disorder of endogenous metabolites. Extensive studies have illuminated that a variety of natural products protected against DKD by regulating diverse signals, such as inflammation pathways [IκB/NF-κB, JAK/STAT, MAPK and NOD-like receptor family pyrin domain containing 3 (NLRP3)], oxidative stress pathways (AGEs/RAGE and Keap1/Nrf2), energy/autophagy pathways (PI3K/AKT/mTOR, AMPK and endoplasmic reticulum stress) and fibrosis pathways (TGF-β/Smad and Wnt/β-catenin) [45–47].

Previous publications were systematically retrieved from multiple electronic databases, including Web of Science, PubMed, ScienceDirect, Wiley, and Nature Springer by using keywords, including “diabetic kidney disease” or “diabetic nephropathy” or “DKD” or “DN” and “natural product*” or “natural medicine*” or “traditional Chinese medicine*” or “herb*” or “phytochemical” or “Chinese herbal medicine*” or “Chinese medicinal herbs” or “Chinese traditional patent medicine”. This review further systematically summarizes these signalling pathways that might be associated with multiomics-mediated mechanisms. These findings expand our understanding of multiomics-based downstream signalling pathways and therapeutic effects of natural products on DKD and provide valuable information for the clinical application of natural products. This review presents a concept-driven therapeutic strategy for DKD treatment and management.

## DKD

DKD is a prevalent and severe complication of diabetes mellitus, linked to higher rates of morbidity and mortality [48]. DKD is clinically recognized by ongoing albuminuria, specifically an albumin-to-creatinine ratio exceeding 30 mg/g of creatinine, paired with gradual deterioration of kidney function [49]. This condition is a microvascular complication arising from diabetes and is marked by hyperfiltration and expansion of mesangial matrix. These changes contribute to kidney hypertrophy, glomerular basement membrane (GBM) thickening, and subsequent injuries to both podocytes and glomerulus, along with damage to renal tubules [49, 50]. Ultimately, these processes culminate in glomerulosclerosis and tubulointerstitial fibrosis [50]. The onset of DKD is due to complex interaction of various structural, hemodynamic, and inflammatory factors that drive both onset and progression of condition [51, 52]. While standard treatments that involve rigorous management of blood sugar and blood pressure are commonly employed, they fall short of halting advancement of DKD toward ESRD and related mortality [53]. DKD is caused by impaired metabolic, fibrotic, hemodynamic, and inflammatory processes [50, 54]. In response to this challenge, extensive research has been dedicated to exploring and substantiating potential benefits of natural products in both experimental and clinical settings [45].

Multiple molecular mechanisms have highlighted that prolonged and persistent hyperglycemia levels led to a variety of cellular damage processes in the kidneys that ultimately resulted in glomerulosclerosis and tubulointerstitial fibrosis [7]. Intracellular glucose levels exceed glycolytic capacity, diverting carbon flux to four major stress pathways: polyol signaling pathway, AGE formation, protein kinase C (PKC) activation, and hexosamine biosynthesis pathway. Each pathway exacerbates oxidative stress, disrupts redox balance, and alters gene transcription and regulation, creating a vicious cycle process. Chronic PKC activation in glomerular endothelial cells increases the levels of vascular endothelial growth factor A and endothelin 1 that damage filtration barrier and promote albumin leakage into glomeruli, which in turn damages renal tubules. Mesangial cells accelerate the excessive accumulation and deposition ECM components and glomerulosclerosis by upregulating expression of TGF-β and connective tissue growth factor, thus affecting AGE levels [55]. Due to persistent exposure to reactive oxygen species, podocytes suffer from actin cytoskeleton disruption and detachment [23]. Hyperglycemic environment also mediates renal tubular epithelial cell damage. Mitochondrial overload increases oxidative stress production, while hexosamine pathway regulates key transcription factors, leading them to pro-fibrotic gene programs [56]. Impaired autophagy flux results in the accumulation of damaged mitochondria and protein aggregates. Functionally impaired renal tubules secrete chemokines that recruits macrophages and T cells, which form an exacerbating the vicious cycle of inflammation and interstitial fibrosis [57]. Hyperglycemic milieu also reprograms resident immune cells. High levels of glucose and AGE cause macrophages to polarize into an M1-like pro-inflammatory phenotype, and persistent ROS impair their phagocytic capacity [57]. Single-cell RNA sequencing study suggested that aberrant immune and matrix microenvironments co-evolve, reinforcing metabolic memory formed from early glycemic fluctuations [19].

## Multiomics in health and diseases

Systems biology-based on multiomics, including genomics, transcriptomics, proteomics and metabolomics, is a cross-application of various high-throughput screening platforms [58]. Multiomics plays an important role in improving human health and disease research. Multiomics integrated analysis helps us to elucidate molecular mechanisms of cell biology and human diseases [59, 60]. In addition, multiomics technology provides a complete research method for revealing interactions of intracellular and intercellular molecular mechanisms related to occurrence, development and pathogenesis of human diseases [59–61]. Multiomics has been widely used in a variety of diseases including DKD to assess risk of disease progression, identify diagnostic biomarkers, reveal pathological mechanisms of diseases, diagnose and prognose diseases and discover therapeutic targets [61–65].

## Natural products improved DKD by regulating multiomics signalling

**Fig. 1:**
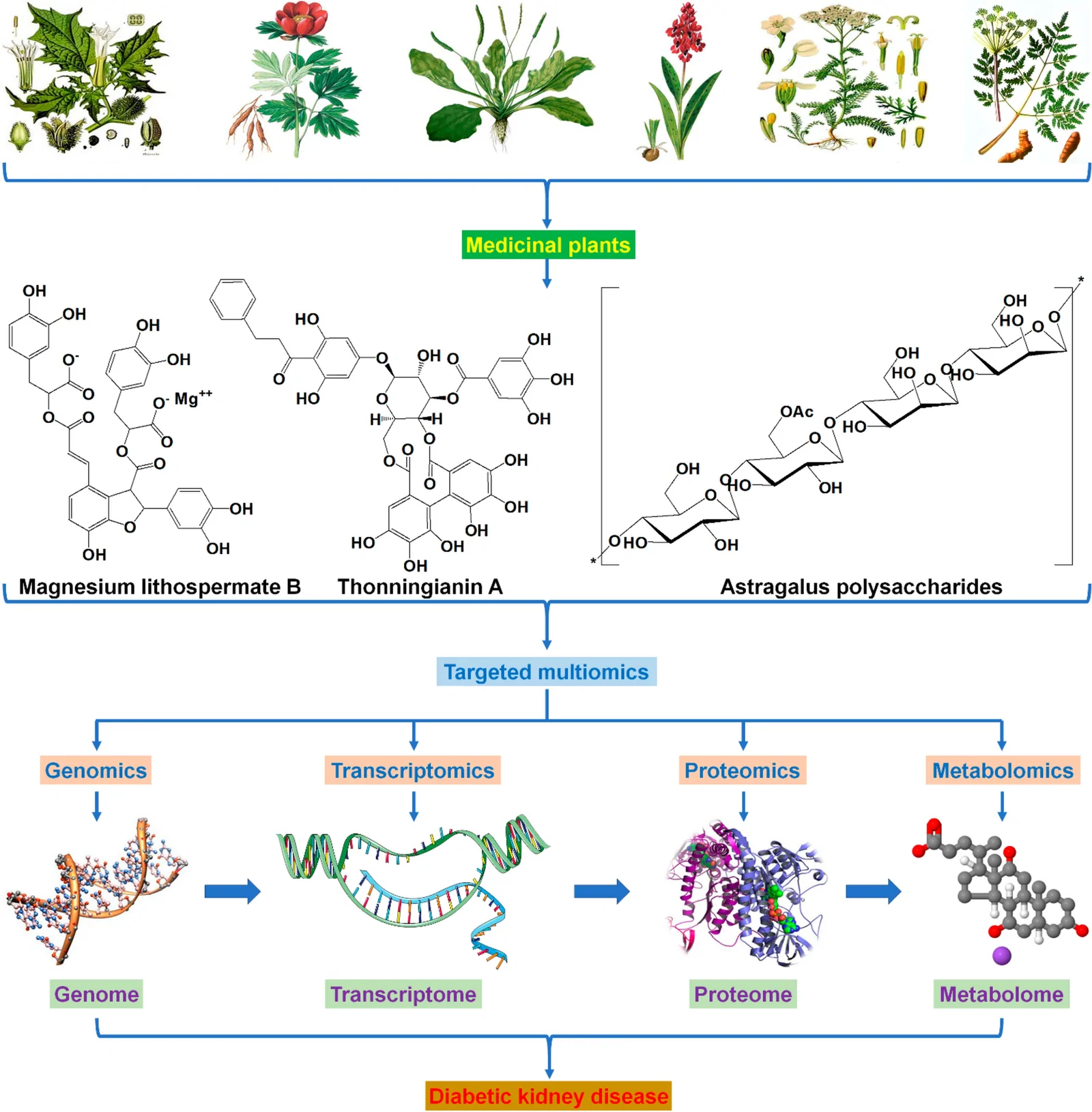
Natural products improve DKD by targeting multiomics-mediated novel molecular mechanisms. Natural products and their active components, such as magnesium lithospermate B (MLB), Thonningianin A and Astragalus polysaccharides, regulated change of genome, transcriptome, proteome and metabolome based on genomics, transcriptomics, proteomics and metabolomics in DKD

Extensive research has demonstrated that natural products, including TCM, can ameliorate DKD by improving kidney function and reducing kidney damage [66–69]. Increasing research has shown that multiomics techniques have been used to identify therapeutic targets and elucidate molecular mechanisms by which natural products improve DKD [70, 71] (Fig. 1).

### Natural products ameliorated DKD by regulating GM dysbiosis

Accumulated studies have shown that GM dysbiosis is closely related to development and progression of CKD, including DKD [72–74]. GM has emerged as a rapidly expanding area of research. Numerous studies conducted in both patients and animal models have demonstrated a strong association between GM and onset and development of metabolic disorders [75]. An imbalance in GM can exacerbate kidney conditions [76]. Increasing publications have suggested that gut-derived metabolites are closely related to kidney function and regulating microbial metabolites have potential to enhance kidney function [77, 78]. Previous studies have shown that various natural products retard DKD by reshaping GM dysbiosis (Table 1).

**Table 1:** Therapeutic effects of TCM formulas, extracts and compounds on DKD by targeting multiomics

Multiomics	Drugs	Resource	Animals/cells	Molecular mechanisms	References
Genomics, transcriptomics and metabolomics	Qitu Qushi formula	TCM formula	Patients and *db/db* mice	Regulated GM dysbiosis, tryptophan metabolism and increased IPA levels and enhanced renal Sirt1/FoxO1 pathway	[83]
Genomics	Jin Gui Ren Qi Pill	TCM formula	Patients	Increased *Prevotella_7* abundance, enhanced carbohydrate metabolism and reduced IL-2 levels	[84]
Metagenomics	*Ramulus Mori* alkaloids	*Ramulus Mori*	C57BL/6 J mice, MPC5 cells	Restored GM dysbiosis	[98]
Metagenomics	Magnesium lithospermate B	*Salvia miltiorrhizae* radix	SD rats, mouse GMCs	Regulated GM dysbiosis	[99]
Metagenomics	Thonningianin A	*Penthorum chinense* Pursh	C57BL/6 mice	Regulated GM dysbiosis	[101]
Metagenomics	Polysaccharides	*Cordyceps cicadae*	SD rats	Reshaped GM dysbiosis	[110]
Metagenomics	Polysaccharides	*Bupleurum Chinese* DC	C57BL/6 mice	Reshaped GM dysbiosis	[111]
Transcriptomics	APS	*Astragalus*	*db/db* mice and HG-induced NRK-52E cells	Inhibited lncRNA Gm41268	[115]
Proteomics	Huangkui capsules	*Abelmoschus manihot*(L.) Medik flower	HG-induced HEK293T cells	Regulated VEGF-A by RAP1 signaling and tight junction pathway	[118]
Metabolomics	Germacrone	Ginger family plants	*db/db* mice	Reversed metabolite disorder	[130]
Metabolomics	CAPE	Propolis honeybees	Kunming mice	Regulated metabolite disorder	[131]
Metabolomics	Total glycoside capsule	*Rehmannia glutinosa* Libosch. leaves	*db/db* mice	Regulated metabolite disorder	[132]

*APS,* Astragalus polysaccharides; *CAPE*, caffeic acid phenethyl ester; *HG*, high glucose; *GM*, gut microbiota; *GMCs*, glomerular mesangial cells; *lncRNA*, long non-coding RNA, *MPC5*, mouse podocyte clone−5; *RAP1*, Ras-proximate−1; *SD*, Sprague–Dawley; *VEGF-A*, vascular endothelial growth factor A

Accumulated evidence has shown that TCM formulas ameliorated CKD by regulating GM dysbiosis [79–82]. Increasing studies have highlighted that TCM formulas can improve DKD by regulating GM dysbiosis [83, 84]. QiDiTangShen granules modulated GM composition and reduced abundance of *Lactobacillus, Bacteroides,* and *Lachnospiraceae*_NK4A136_group alongside enriched *Alloprevotella* populations. Moreover, QiDiTangShen modulated serum bile acid composition, suggesting that gut-microbiota-bile acid axis is a critical nephroprotective effects of molecular mechanism [85]. Huangkui capsules, derived from *Abelmoschus manihot* (L.), have been found to modulate gut microbiome in non-obese diabetic mice suffering from DKD [86]. Meng et al. found that Jowiseungki decoction altered bacterial composition, including Alphaproteobacteria group and various genera, such as *Acetatifactor, Butyricicoccus, Kerstersia*, *Peptococcus*, and *Coriobacteriaceae_UCG-002* in the streptozotocin (STZ)-induced DKD mice [87]. Bekhogainsam decoction has potential to quell inflammation and restore gut balance in DKD rodents. This is accomplished by boosting proliferation of beneficial bacteria, such as those from *Clostridiales* and *Peptococcus* groups, and diminishing prevalence of *Actinobacteria*, as noted in a previous study [88]. Zicuiyin Convention promotes kidney health and corrects intestinal bacterial imbalances [89]. This positive outcome is thought to be due to regulation of various GM, including *Ruminococcaceae_UCG-014*, *Allobaculum*, *Unclassified_f_Lachnospiraceae Alloprevotella*, and *Bacteroides* [89]. San-Huang-Yi-Shen capsule (SHYS) has been used to manage various CKD, such as DKD, immunoglobulin A nephropathy, and chronic renal failure [90]. Analysis through 16S rRNA sequencing revealed that SHYS influenced beta diversity of GM in rats with DKD. The mechanism of SHYS in DKD may be linked to enhancement of GM, which modulates arginine biosynthesis, tricarboxylic acid cycle, tyrosine metabolism, and arginine and proline metabolism [90]. Tangshen formula, a classic Chinese herbal prescription, has demonstrated its ability to mitigate kidney damage, reshape gut microbial makeup, and lower levels of metabolic endotoxemia and lipopolysaccharide (LPS) in DKD rats [91]. Furthermore, Qing-Re-Xiao-Zheng formula also had a favorable impact on urinary albumin levels, colon mucosal integrity, gut imbalances, and gut-originated LPS in a DKD mouse model [92].

Natural compounds are an important source of new drugs [93–97]. *Ramulus Mori* alkaloids are potent components derived from *Ramulus Mori* and may combat diabetes and obesity by promoting glucose and lipid metabolism [98]. Furthermore, after treatment with *Ramulus Mori* alkaloids, the composition of gut bacteria shifts, with a notable increase in *Dubosiella* and higher levels of serum pentadecanoic acid [98]. Magnesium lithospermate B (MLB), a key element in Danshen water extracts, has shown considerable reductions in urinary albumin to taurocholic acid ratio over 24 h when administered to DKD rats [99]. Despite its remarkably low bioavailability, MLB exhibits a notable oral activity. This suggests that its beneficial effects on kidney damage might be linked to its influence on the gut microbiome and bile acid metabolism [100] (Fig. 1). Thonningianin A (TA) is a key constituent of herbal remedy *Penthorum chinense*. Recent pharmacological research has validated efficacy of TA in combating oxidation, inflammation, and fibrosis [101]. These findings demonstrated that TA significantly reduced blood glucose levels, decreased 24-h urinary total protein, and improved renal dysfunction in DKD mice [101]. Moreover, it dramatically reduces renal damage and interstitial fibrosis while suppressing renal inflammation [101]. TA ameliorated intestinal barrier dysfunction by upregulating critical junctional proteins, such as occludin and zonula occludens-1 [101] (Fig. 1). This, in turn, decreased levels of lipopolysaccharide in both feces and serum of DKD mice. A 16S rRNA sequencing analysis revealed that TA ameliorated GM dysbiosis by reducing abundance of gram-negative bacteria, specifically Proteobacteria and *Escherichia-Shigella* taxa [101].

Rapidly increasing publications have shown that many natural polysaccharides exhibit a wide range of pharmacological activities [102–105]. Recent studies have shown that natural polysaccharides improve DKD by regulating GM dysbiosis [106–108]. Administering polysaccharide derived from *Moutan Cortex* demonstrated a significant reduction in hyperglycemia and kidney damage in DKD rats [109]. Furthermore, it is crucial in repairing gut microbiome, restoring integrity of intestinal barrier, and increasing concentration of short-chain fatty acids [109]. Studies have revealed that *Cordyceps cicadae* polysaccharides increase insulin sensitivity and glucose tolerance in diabetic rats [110]. In addition, *Cordyceps cicadae* polysaccharides can balance gut microbiome by increasing presence and growth of beneficial bacteria [110]. *Bupleurum* polysaccharides were extracted from *Bupleurum chinense* DC. effectively alleviates STZ-induced DKD [111]. These polysaccharides reduced the levels of blood glucose, serum creatinine and urine albumin [111]. Additionally, GM showed a shift toward greater diversity and an increase in protective bacteria. Improvements were also observed in gut barrier along with a decrease in inflammatory responses in both kidneys and colon [111]. These results underscore role of GM modulation and inflammation reduction as key mechanisms through which *Bupleurum* polysaccharides exert protective effects against DKD in mice [111].

### Natural products retarded DKD by regulating aberrant expression of ncRNAs

The ncRNAs play essential roles in regulating development and progression of diverse diseases [112, 113]. Increasing research suggested that natural products improve DKD by enhancing keap1/Nrf2 signalling pathway via regulating ncRNAs [114]. Recent study identified a novel long non-coding RNA (lncRNA), Gm41268, as cis-regulatory target gene prolactin receptor (PRLR). Astragalus polysaccharides (APS) downregulated fibronectin, TGF-β, and collagen IV levels by regulating autophagy via downregulating protein levels of p62 and p-mTOR while elevating ratio of LC3 II/I [115]. Moreover, in vitro experiments showed that APS restored high glucose (HG)-induced Gm41268 and PRLR overexpression [115] (Fig. 1). Reduced Gm41268 downregulated PRLR expression, restored autophagy, and attenuated renal fibrosis. By contrast, APS effects were enhanced by suppressing PRLR [115] (Table 1).

miRNAs represent a category of non-coding RNAs that interact with 3' untranslated region (3'UTR) of specific mRNAs, enabling post-transcriptional regulation of disease-associated signaling networks [116]. Emerging evidence has identified dysregulated miRNAs as pivotal modulators of DKD pathogenesis through fibrotic and inflammatory pathway interactions [116]. Consequently, miRNAs are promising candidates for therapeutic interventions in DKD [116]. Triptolide showed a substantial reduction in albuminuria and improved glomerulosclerosis in rats with DKD [117]. In HG-treated human renal mesangial cells (HRMCs) and kidneys of diabetic rats, miR-137 levels were notably lower, but triptolide helped boost them [117]. Furthermore, overexpression of miR-137 mimicked benefits of triptolide, whereas its inhibition led to an increase in ECM protein accumulation [117].

### Natural products ameliorated DKD by regulating altered proteome

Huangkui capsules (HKC) were used as a clinical therapeutic drug for DKD patients [118]. Combined HG-stimulated HEK293T cells and network pharmacology analysis identified a total of 4 known targets and 86 potential unknown targets of HKC [118]. The above targets were verified by using urinary proteomics of DKD patients before and after HKC treatment. The results showed that quercetin and palmitic acid in HKC mitigated kidney damage in DKD patients by vascular endothelial growth factor A (VEGF-A) through Ras-proximate−1 (RAP1) signaling and tight junction pathways [118].

### Natural products retarded DKD by regulating dysregulation of metabolites

Emerging metabolomics was used for the identification of disease biomarkers, discovery of disease molecular mechanisms, and drug intervention for CKD treatment and renal fibrosis [119–122]. Bekhogainsam decoction (BHID) was prepared from *gypsum fibrosum*, *Anemarrhenae rhizome*, *Ginseng radix* et rhizome, *Glycyrrhizae radix* et rhizome, and polished round-grained rice [123]. BHID effectively halted physiological and serological alterations, structural damage, and renal impairment of mice with STZ-induced DKD [88]. Metabolomic analysis revealed significant shifts in metabolites in BHID treatment [88]. Additionally, Meng et al. identified thirty-six proteins associated with BHID and explored four metabolic pathways, including biosynthesis of valine, leucine, and isoleucine; metabolism of nicotinate and nicotinamide; tryptophan metabolism; and pathways related to alanine, aspartate, and glutamate metabolism [88]. Gandhi capsules (GDC) are preferred clinical treatment for DKD because of their ability to reduce urinary albumin levels and increase glomerular filtration rate [124]. Through metabolomics research, researchers have identified 16 disrupted metabolites as potential biomarkers for DKD [124]. Notably, these metabolites were largely corrected and partially restored to normal levels in patients treated with GDC [124]. The capsule's efficacy in mitigating DKD could be attributed to its ability to correct abnormal metabolic imbalances, such as glycine, tryptophan, valine, leucine, isoleucine metabolism, purine, nitrotoluene, phenylalanine, fatty acid, tyrosine, and bile acid pathways [124]. Consequently, GDC has been proposed as a promising therapeutic option for DKD [124]. Metabolomic and lipidomic analyses have revealed that Chaihuang-Yishen formula exerts a notable therapeutic effect on DKD by effectively curbing levels of various organic toxins, including several uremic toxins and glucuronides, while also restoring deficits in phospholipids, particularly sphingomyelins [125]. Both Chaihuang-Yishen formula and fosinopril demonstrate a protective effect on renal health in context of DKD [125]. The enhancement of disrupted metabolic and lipid profiles, characterized by reduction of uremic toxins and glucuronides along with normalization of phospholipid levels, likely underlies mechanisms through which these treatments mitigate progression of DKD [125]. Shenyan Kangfu tablets are a traditional formulation that has been documented in *Synopsis of Golden Chamber* [126]. Metabolomic studies have revealed that the most crucial pathways through which Shenyan Kangfu tablets addresses DKD are biosynthesis of unsaturated fatty acids and metabolism of starch and sucrose [126].

## Natural products improving DKD by regulating multiomics-associated signalling pathways

Accumulated publications have shown that natural products improve DKD by regulating diverse downstream molecular mechanisms, such as PI3K/GSK-3β/Nrf2/TGF-β/Smad, toll-like receptor 4 (TLR4)/NF-κB/NLRP3, peroxisome proliferator-activated receptor γ and liver X receptor α signalling pathways [127–129].

An increasing number of studies have shown that various natural products retard DKD by regulating metabolite disorders (Table 1). Germacrone, a natural bioactive compound from medicinal plants of ginger family, has anti-inflammatory and antioxidant properties [130]. Germacrone enhances kidney health in diabetic mice by modulating associated upregulation of metabolites, such as oleic acid and lithocholic acid, and decline of stearic acid [130]. Caffeic acid phenethyl ester (CAPE) is a key component in propolis honeybee production [131]. Metabolomics analysis showed that significant alterations in serum levels of LysoPC, 2,3-dinor-8-iso-PGF2a, phosphatidylserine, diglycerides, and arachidonic acid were identified as potential biomarkers in cadmium-induced DKD in mice, and CAPE treatment restored these levels to near-normal ranges [131]. The total glycoside capsule was formulated from phenylethanol glycoside group present in Dihuang leaves and has been introduced in China as a Class II Chinese medicine aimed at treating renal disorders [132]. The primary ingredient in this capsule is acetonide. Research suggests that acetonide delay DKD progression by ameliorating renal histopathology [132]. In kidneys of db/db mice, amino acid metabolism was disrupted, but this imbalance could be rectified by acetonide, affecting levels of tryptophan, glutamine, cysteine, leucine, threonine, proline, phenylalanine, histidine, serine, arginine, and asparagine, as indicated by targeted metabolomics [132].

### Therapeutic effects of natural products on DKD patients by multiomics-associated mechanisms

Increasing evidence has demonstrated that Chinese traditional patent medicines are usually used to treat patients with DKD [133, 134]. A TCM formula Qitu Qushi formula displayed therapeutic effect in DKD [83]. This formula significantly improved renal function and modulated GM composition in 30 DKD patients treated for 6 months. Multi-omics study showed that QTQSF regulated GM tryptophan metabolism and increased indole−3-propionic acid (IPA) levels that were related to enhancing renal Sirt1/FoxO1 pathway in *db/db* mice [83]. A double-blind randomized table method involving 30 DKD patients showed that a 4-week therapy regimen of combining Jin Gui Ren Qi Pill with *Bifidobacterium bifidum* tetragonum tablets outperforms standard Western medical care in alleviating symptoms and lowering urinary albumin-to-creatinine ratio (UACR) in patients with DKD [84]. This combination therapy increased *Prevotella_7* abundance in the faeces of DKD patients, which was associated with enhancing carbohydrate metabolism by GM [84].

### Renoprotective effects of natural products on DKD by multiomics-associated signalling pathways

**Fig. 2:**
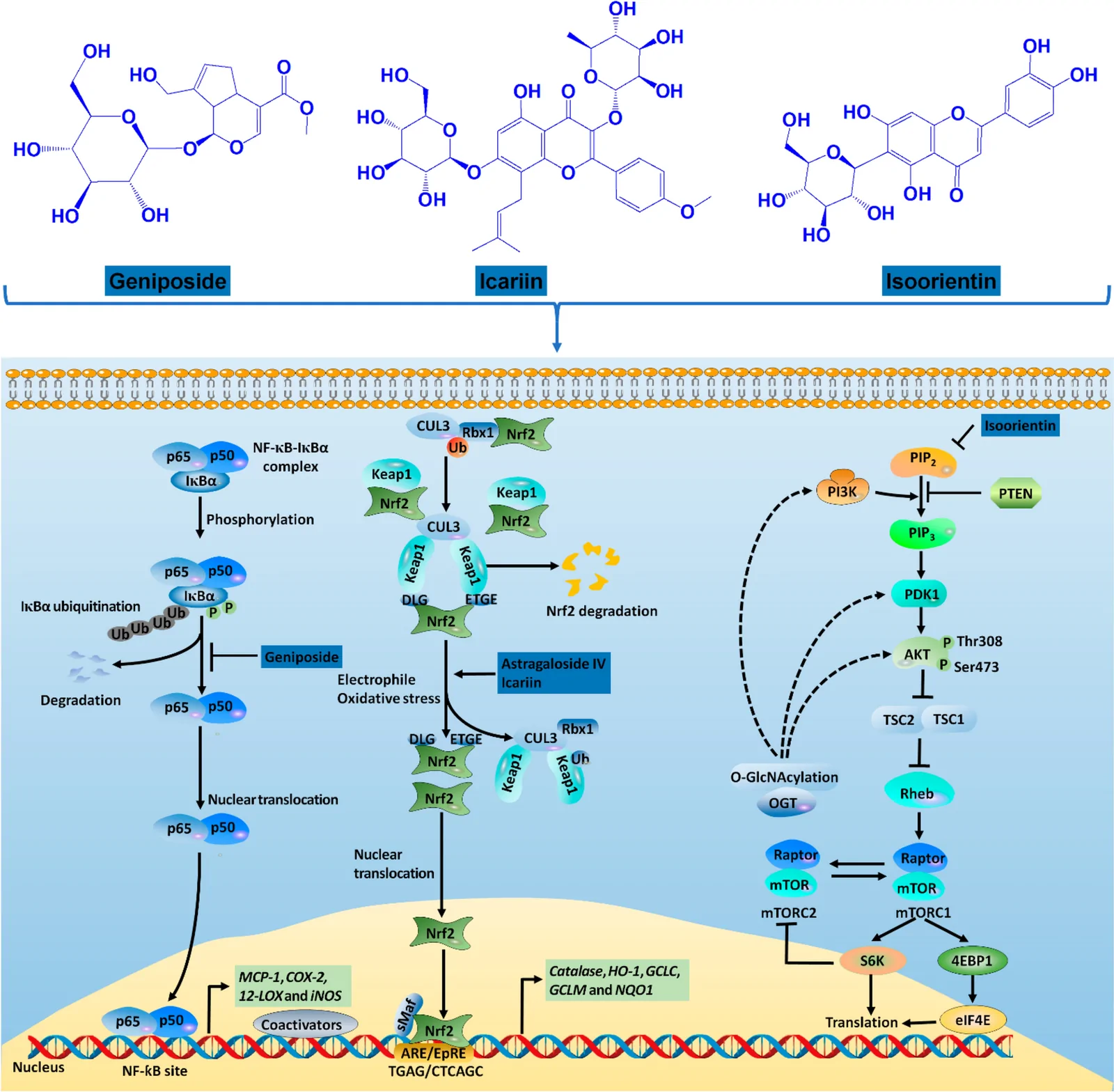
Natural products improved DKD by regulating IκB/NF-κB, Keap1/Nrf2 and PI3K/AKT/mTOR pathways. Natural products, such as geniposide, astragaloside IV, icariin and isoorientin, improved renal functions and ameliorated DKD by modulating several inflammation-associated key signals, such as IκB/NF-κB, Keap1/Nrf2 and PI3K/AKT/mTOR pathways

**Fig. 3:**
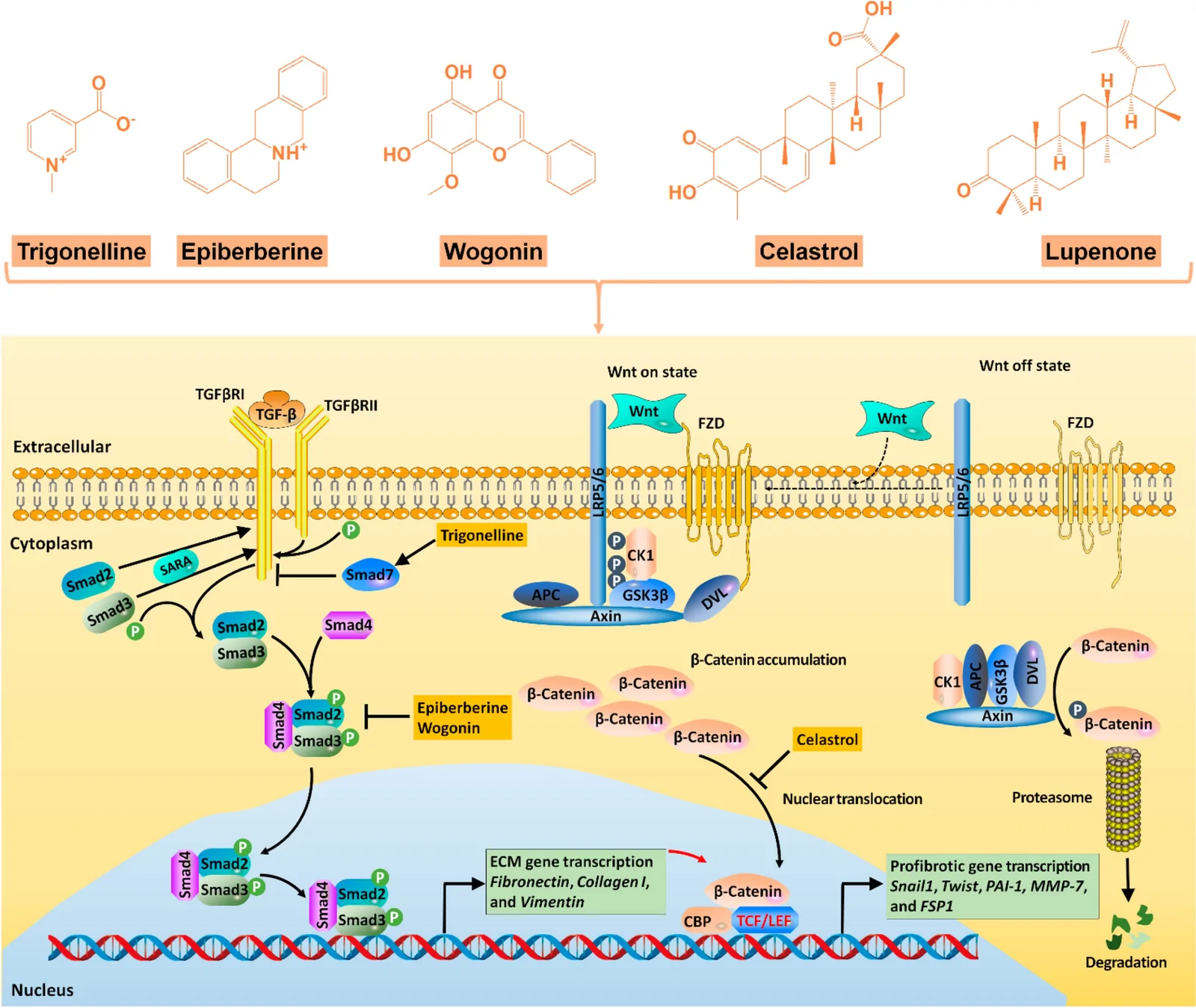
Natural products improved DKD by inhibiting TGF-β/Smad and Wnt/β-catenin pathways. Natural products, such as trigonelline, epiberberine, wogonin, celastrol and lupenone, protected against DKD by modulating several critical signals, including TGF-β/Smad and Wnt/β-catenin pathways

Natural products have garnered increasing attention globally because of their potential as important treatments and as rich resources for novel medicines [135–139]. Natural products are becoming increasingly prevalent because they are available from various sources at relatively low prices and have fewer adverse effects [140–145]. They enhance DKD by regulating a variety of molecular mechanisms, such as AGE/RAGE, IκB/NF-κB, TGF-β/Smad, Wnt/β-catenin, AMPK, MAPK, NLRP3 inflammasome, JAK/STAT and autophagy signaling pathways (Figs. 2 and 3 and Tables 1, 2, 3).

**Table 2:** Therapeutic effects of TCM formulas and extracts on DKD by targeting diverse signalling pathways

Drugs	Resource	Animals/cells	Molecular mechanisms	References
Huangkui capsule	*Abelmoschus manihot* (L.) Medik flower	SD rats	Inhibited NLRP3 inflammasome	[86]
APS	*Astragalus*	*db/db* mice and NRK-52E cells	Regulated autophagy pathway	[115]
*Salvia miltiorrhiza* extracts	*Salvia miltiorrhiza*	SD rats	Suppressed Wnt/β-catenin pathway	[180]
Total glycosides	*Rehmannia glutinosa* Libosch	SD rats, mouse GMCs	Suppressed Wnt/β-catenin pathway	[181]
*Physalis peruviana* fruit extract	*Physalis peruviana* L	Albino rats	Inhibited AMPK/mTOR pathway	[192]
Korean red ginseng extract	Korean red ginseng	SD rats	Regulated autophagy pathway	[193]
RPF	*Radix astragali*	C57BL/6 mice, podocytes	Inhibited PI3K/AKT/mTOR pathway	[224]
Total glucosides	*Paeonia alba*	Munich-Wistar rats	Inhibited ER stress	[228]
Total flavones	*Abelmoschus manihot* flowers	Rats, HK-2 and HRMC cells	Inhibited ER stress	[230]

*AGE*, advanced glycation end products; *AKT*, protein kinase B; *AMPK*, adenosine 5'-monophosphate-activated protein kinase; *APS*, Astragalus polysaccharides; *ER*, endoplasmic reticulum; *HK-2*, human kidney-2; *HRMC*, human renal mesangial cells; *ICR*, institute for cancer research; *mTOR*, mammalian target of rapamycin; *NLRP3*, NOD-like receptor family pyrin domain containing 3; *PI3K*, phosphoinositide 3-kinases; *RAGE*, receptor for advanced glycation end products; *SD*, Sprague–Dawley

**Table 3:** Therapeutic effects of the active compounds on DKD by targeting diverse signalling pathways

Compounds	Resources	Animals/cells	Molecular mechanisms	References
Triptolide	*Tripterygium wilfordii* Hook F	SD rats, HRMCs	Regulated miRNA-137/Notch homolog 1 pathway	[117]
Ellagic acid and gallic acid	*Syzygium cumini (L.) Skeels*	HEK293 cells	Inhibited AGE/RAGE pathway	[149]
Dieckol	*Ecklonia cava*	Mouse GMCs	Inhibited AGE/RAGE pathway	[152]
Vanillin	*Vanilla planifolia*	SD rats	Blocked AGE/RAGE pathway	[153]
Geniposide	*Gardenia jasminoides* Ellis	*db/db* mice	Inhibited AGE/RAGE pathway	[154]
Catalpol	*Rehmanniae Radix*	KK-Ay mice, mouse glomerular endothelial cells	Blocked AGE/RAGE pathway	[155]
Loganin	*Cornus officinalis*	C57BL/6 J mice	Inhibited AGE/RAGE pathway	[156]
Hesperetin	Sweet orange and lemon	SD rats	Inhibited AGE/RAGE pathway	[157]
Geniposide	*Gardenia jasminoides* Ellis fruit	STZ-induced diabetic rats	Inhibited IκB/NF-κB pathway	[159]
Astragaloside IV	Huangqi decoction	SD rats, MPC5 cells	Enhancing Keap1/Nrf2 pathway	[162]
Icariin	*Herba Epimedii*	SD rats	Enhancing Keap1/Nrf2 pathway	[164]
Trigonelline	*Trigonella foenum-graecum* L	*db/db* mice	Inhibited TGF-β/Smad pathway	[169]
Lupenone	Banana peel	*db/db* mice	Inhibited TGF-β/Smad pathway	[170]
Silymarin	*Silybum marianum* seeds	SD rats, MPC5 cells	Blocked TGF-β/Smad pathway	[171]
Epiberberine	*Rhizome coptidis*	*db/db* mice, GMCs	Inhibited TGF-β/Smad2 pathway	[172]
Wogonin	*Scutellaria baicalensis*	C57/BL mice, GMCs, SV40	Blocked TGF-β/Smad3 pathway	[173]
Celastrol	*Tripterygium wilfordii* Hook. f	*db/db* mice, MPC5 cells	Inhibited Wnt/β-catenin pathway	[182]
Asiatic acid	*Cyclocarya paliurus*	SD rats, HK-2 cells	Regulated autophagy pathway	[195]
DMDD	*Averrhoa carambola* L	C57BL/6 mice, HK-2 cells	Regulated autophagy pathway	[197]
Ferulic acid	*Ferula asafoetida* L., and *Anthemis nobilis* L	C57BL/6 mice	Regulated autophagy pathway	[198]
CMP	*Cordyceps militaris*	C57BL/6 mice	Regulated autophagy pathway	[199]
Kaempferol	Tea leaves, broccoli, hazelnuts, propolis, grapefruit	*db/db* mice	Inhibited AMPK/mTOR pathway	[209]
Corilagin	Many plants	C57BL/6 mice, MPC5 cells	Regulated AMPK/SIRT1 pathway	[211]
Marein	*Coreopsis tinctoria* Nutt	db/db mice, HK-2 cells	Regulated AMPK pathway	[212]
Salvianolic acid B and tanshinone IIA	*Salvia miltiorrhiza*	SD rats	Inhibited PI3K/AKT/NF-κB pathway	[216]
Quercetin	Fruits and vegetables	*db/db* mice, MPC5 cells	Regulated autophagy pathway	[217]
Isoorientin	Fenugreek	C57BL/6 J mice, MPC5 cells	Inhibited PI3K/AKT/mTOR pathway	[221]
Rutin	*Sophora japonica* and *Astragalus membranaceus*	*db/db* mice, human renal glomerular endothelial cells	Inhibited PI3K/AKT/mTOR pathway	[222]
Paeoniflorin	*Paeonia lactiflora* Pall	C57BL/6 J mice, MPC5 cells	Inhibited PI3K/AKT pathway	[223]
Chrysin	Propolis and mushrooms	*db/db* mice, MPC5 cells	Inhibited ER stress	[229]
Arctigenin	*Fructus arctii*	*db/db* mice, HK-2 cells	Inhibited ER stress	[233]
Apigenin	Fruits, nuts, tea, and soy	Albino Wistar rats	Inhibited MAPK/NF-κB pathway	[237]
Morin	Bark of Moraceae	Albino Wistar rats	Inhibited MAPK pathway	[240]
Hirudin	Salivary glands of leeches	SD rats	IκB/NF-κB pathway	[232]
20(R)-Rg3	*Panax ginseng* C. A	C57BL/6 mice	Inhibited MAPK/NF-κB pathway	[242]

*AGE*, advanced glycation end products; *AKT*, protein kinase B; *AMPK*, adenosine 5'-monophosphate-activated protein kinase; *APS*, Astragalus polysaccharides; *CMP*, *Cordyceps militaris* polysaccharides; *DMDD*, 2-dodecyl-6-methoxycyclohexa-2,5-diene−1,4-dione; *ER*, endoplasmic reticulum; *GMCs*, glomerular mesangial cells; *HK-2*, human kidney-2; *HRMCs*, human renal mesangial cells; *IκB*, inhibitor of kappa B; *Keap1*, kelch-like ECH-associated protein 1; *MAPK*, mitogen-activated protein kinases; *MPC5*, mouse podocyte clone−5; *mTOR*, mammalian target of rapamycin; *NF-κB*, nuclear factor kappa B; *NLRP3*, NOD-like receptor family pyrin domain containing 3; *Nrf2*, nuclear factor erythroid 2-related factor 2; *PI3K*, phosphoinositide 3-kinases; *RAGE*, receptor for advanced glycation end products; *SD*, Sprague–Dawley; *Smad*, suppressor of Mothers against decapentaplegic; *STZ*, streptozotocin; *TGF-β*, transforming growth factor β

#### Natural products inhibited DKD by suppressing AGE/RAGE signaling pathway

AGE is involved in the pathogenesis of diabetic complications, and formation of irreversible AGE promotes deposition of ECM such as collagen in glomeruli via RAGE and abnormal glomerular remodeling in kidney [146]. Targeting AGE/RAGE axis by suppressing renal deposition of AGE and blocking receptor signaling has emerged as a pivotal strategy for DKD prevention [146]. Several studies have demonstrated that a variety of natural products improve DKD by regulating AGE/RAGE signaling pathway (Tables 2 and 3).

Dang-Gui-Bu-Xue decoction was formulated with a blend of *Astragalus membranaceus* (Fisch.) Bge., *Angelica sinensis* (Oliv.) Diels [147]. it reduces renal oxidative stress induced by high-fat diet and STZ in diabetic mice by targeting AGE/RAGE pathway [147]. Huang-Lian-Jie-Du decoction was formulated using *Gardenia jasminoides*, *Scutellaria baicalensis*, *Phellodendron amurense*, and *Coptis chinensis* [148]. Studies have demonstrated that following its treatment, there is a notable increase in glyoxalase I expression and considerable reduction in RAGE expression in db/db mice [148]. This evidence demonstrates that this formula may improve diabetes by effectively blocking AGE/RAGE pathway [148].

*Syzygium cumini* homeopathic formulation derived from *Syzygium cumini* (L.) Skeels seeds contains ellagic acid and gallic acid as its primary phytochemicals [149]. Mechanistically, they inhibit RAGE promoter activity [149]. The leaves of *Hippophae rhamnoides* L. (sea buckthorn leaves; SBL) are preferred therapeutic agents for managing diverse ailments [150]. Histological changes and UACR were ameliorated in db/db mice after oral administration of SBL extract, and accumulation of AGE in glomeruli was inhibited [150]. Moreover, SBL helped mitigate oxidative stress in diabetic kidneys by cutting down AGE, which in turn reduced renal damage. Based on these findings, SBL extract holds promise as a therapeutic option for managing diabetic kidney complications associated with AGE [150].

Dieckol, a compound derived from *Ecklonia cava*, can inhibit DKD [151]. Dieckol attenuates renal damage induced by methylglyoxal in mouse glomerular mesangial cells (GMCs) by suppressing AGE-RAGE Interaction [152]. Vanillin is a naturally occurring phenolic compound found in nature. It is the primary component of *Vanilla planifolia* [153]. Vanillin ameliorated fibrosis, inflammatory responses, oxidative stress, and AGE production in DKD rats [153]. Geniposide, main bioactive compound in *Gardenia jasminoides* Ellis, is a natural iridoid glycoside with a variety of therapeutic advantages, including antidiabetic, antioxidant, anti-inflammatory and effects [154]. Geniposide effectively inhibits RAGE expression and inflammatory responses triggered in presence of AGE, matching effects of RAGE siRNA [154]. When administered orally over a long period, geniposide in db/db mice leads to enhanced levels of serum creatinine and proteinuria and reduced pathological changes [154]. Additionally, a dose-dependent decline in renal RAGE expression has been observed [154]. Catalpol is a compound extracted from roots of *Rehmanniae Radix*. It demonstrates therapeutic potential for diabetes mellitus [155]. It directly repairs kidney damage and decreases levels of serum creatinine and urea as well as urine protein, in DKD mice [155]. The compound catalpol reversed endothelial dysfunction and inflammation exacerbated by RAGE overexpression in DKD mice [155]. Loganin is a key bioactive element found in *Cornus officinalis* [156]*.* Loganin reduced AGE levels in serum and kidney tissue, while downregulating mRNA and protein expression of AGE receptors in kidneys of diabetic mice [156]. Hesperidin significantly improves renal function and structural changes in STZ-induced diabetic rats by inhibiting AGE/RAGE axis [157]. Consequently, suppression of AGE formation by natural products serves as a key mechanism for alleviating DKD progression.

#### Natural products inhibited DKD by suppressing IκB/NF-κB and enhancing Keap1/Nrf2 signaling pathways

Oxidative stress and inflammation play a central role in the development and progression of CKD [158]. An earlier study showed that STZ-induced diabetic rats showed an increased levels of serum creatinine and urea, urinary albumin and GBM thickening [159]. However, geniposide improved renal function and GBM changes. Moreover, STZ-induced diabetic rats showed monocytes infiltration levels that positively correlated with GBM severity, increased expression of intercellular adhesion molecule 1, tumor necrosis factor-α, interleukin-1, and interleukin-6 [159]. STZ-induced diabetic rats showed upregulation expression of NF-κB p65, IKKα, and p-IκBα in renal tissue. However, geniposide treatment reversed all changes in renal inflammation and IκB/NF-κB pathway in STZ-induced diabetic rats [159] (Fig. 2). Recent study showed that geniposide attenuated DKD mice by AMPK/SIRT1/NF-κB pathway [160].

Nrf2 and its endogenous inhibitor Keap1 are ubiquitous, evolutionarily conserved intracellular defense mechanisms that can combat oxidative stress and inflammatory responses [161]. Recent studies have shown that astragaloside IV (AS-IV) promotes dissociation of Nrf2 and Keap1/Nrf2 interaction by competitively binding to amino acid sites in Keap1 [162] (Fig. 2). HG-induced podocytes showed podocyte apoptosis and Nrf2 expression downregulation accompanied by increasing ROS production [162]. AS-IV treatment decreased ROS production. However, Nrf2 inhibitor attenuated beneficial effects of AS-IV. Similar results suggested that diabetic mice showed renal injury and decreased Nrf2 expression. By contrast, AS-IV restored Nrf2 expression [162]. Flavonoid icariin from *Herba epimedii* exert anti-inflammatory and anti-fibrotic effects [163]. Icariin treatment could activate Nrf2, inhibit NLRP3 and degrade Keap1 by Sesn2-dependant mitophagy in STZ-induced rats [163]. Sesn2 siRNA showed that icariin-induced NLRP3 suppression and mitophagy were partly abolished in HG-treated MPC5 cells [163]. These findings suggest that icariin restored podocyte injury by inhibiting NLRP3 activation through Sesn2-mediated mitophagy via Keap1/Nrf2 signalling pathway in DKD. Moreover, icariin treatment also inhibited ECM accumulation and attenuated DKD through suppressing oxidative stress via G protein-coupled estrogen receptor induced p62-dependent Keap1 degradation and Nrf2 activation [164].

#### Natural products suppressed DKD by inhibiting TGF-β/Smad signaling pathway

TGF-β/Smad signaling pathway is pivotal in DKD and renal fibrosis [165, 166]. This signaling system manipulates DKD in various ways through its ligands, receptors, and subsequent Smad molecules [165]. During renal fibrosis and inflammation triggered by diabetes, the latent forms of TGF-β1 and Smad7 play defensive roles, whereas active forms of TGF-β1 and Smad3 contribute to disease progression (Fig. 3). Buyang Huanwu decoction mitigated effects of STZ-induced diabetic glomerulosclerosis and tubulointerstitial damage, while also leading to a decrease in renal inflammation and fibrosis [167]. At a mechanistic level, this formula was found to inhibit activation of TGF-β1/Smad3 pathway in DKD and restore renal Smad7 levels [167]. Fuxin granules are composed of a finely ground blend of seven different herbs, namely *Scrophularia ningpoensis* Hemsl, *Rheum palmatum* L., *Ramulus Euonymi*, *Astragalus propinquus* Schischkin, *Alisma plantago-aquatica* Linn, *Anemarrhena asphodeloides* Bunge, and *Cuscuta chinensis* Lam [168]. Fuxin granules exhibits multifaceted therapeutic effects in DKD by modulating TGF-β1/Smad pathway, leading to a considerable reduction in TGF-β1 expression within glomeruli [168]. Furthermore, Fuxin granules significantly inhibited total protein levels of TGF-β1 and Smad2/3, as well as mRNA levels of phosphorylated Smad2 and Smad3 [168].

Several studies have suggested that diverse natural products retard DKD by inhibiting TGF-β/Smad signaling pathway [70] (Tables 2 and 3). Trigonelline is a compound extracted from fenugreek, known as *Trigonella foenum-graecum* L. [169]. Trigonelline could control TGF-β1 signaling by promoting Smad7, which effectively blocked transformation of proximal tubular epithelial cells and prevented DKD-induced renal fibrosis [169], and lupenone, a luminance-type pentacyclic triterpenoid found in banana peels, has been identified [170]. Staining techniques and molecular biology assessments indicate that lupenone protects kidney from type 2 DKD by modulating inflammatory response of TGF-β1/Smad/connective tissue growth factor pathway [170]. Silymarin is a flavonoid lignan derived from hull of *Silybum marianum* seeds, a plant belonging to Asteraceae family [171]. Silymarin ameliorated renal lesions in STZ-induced diabetic rats following chronic administration [171]. Furthermore, silymarin nanoliposome therapy significantly dampened TGF-β/Smad signaling pathway, demonstrating kidney protection in rats with DKD [171]. Epiberberine is an isoquinoline alkaloid [172]. Studies in laboratory mice have shown that epiberberine administration can dramatically alleviate metabolic problems linked to diabetes, promote kidney health, and mitigate fibrosis in kidneys of db/db mice [172]. Epiberberine has also been observed to reduce TGF-β1 and Smad2 levels in vitro and in vivo (Fig. 3) [172]. Wogonin, a flavonoid extracted from *Scutellaria baicalensis*, treats DKD by inhibiting oxidative stress and inflammation [173]. Wogonin reduces production of extracellular matrix components triggered by HG levels in kidney and mesangial cells of diabetic mice by inhibiting TGF-β1/Smad3 pathway (Fig. 3) [173].

TGF-β/Smad signaling can influence DKD by increasing or inhibiting levels of microRNAs and lncRNAs associated with Smad3, thus promoting or suppressing DKD progression [165]. A recent study demonstrated that APS alleviated fibrosis by downregulating TGF-β expression DKD by targeting lncRNA Gm41268/PRLR pathway in DKD [115] (Table 1).

#### Natural products attenuated DKD by suppressing Wnt/β-catenin signaling pathway

The Wnt/β-catenin pathway is a key driver of renal fibrogenesis, as evidenced by extensive mechanistic studies [174–176]. Consequently, the inhibition of Wnt/β-catenin signaling could potentially be a powerful therapeutic approach to alleviate kidney fibrosis [177, 178]. Qishen Yiqi dripping pill (QYDP) is a classic blend of TCM [179]. QYDP reduced serum levels of creatinine and urea, inhibited renal enlargement, and improved renal organization in diabetic rats [179]. Moreover, Wnt1 levels markedly decreased in diabetic rats treated with QYDP. Immunostaining and western blotting further confirmed that QYDP decreased β-catenin protein levels [179]. *Salvia miltiorrhiza* extracts (SM) has been found to suppress levels of Wnt4 and β-catenin in kidney tissue and HG-stimulated mesangial cells [180]. *Rehmannia glutinosa* Libosch is a herb commonly used in TCM. Its leaves are a key part of plant [181]. Additionally, total glycosides of *Rehmannia glutinosa* have been successfully developed into a Dihuangye total glycoside capsule, a category II innovative drug approved in China [181]. In vivo studies indicate that they can lower serum levels of creatinine and urea, mitigate renal interstitial fibrosis, and reduce Wnt4 and β-catenin expression, thereby providing renal protection [181]. Several studies have revealed that a variety of natural products retard DKD by suppressing Wnt/β-catenin signaling pathway (Tables 1 and 2). Celastrol, the key active component of *Tripterygium wilfordii* Hook F., is effective in combating renal injury associated with DKD [182]. It lowered blood sugar levels and renal function markers in *db/db* mice, and reduced renal tissue damage [182]. This compound blocks Wnt/β-catenin signaling pathways, such as Wnt1, Wnt7a, and β-catenin (Fig. 3) [182].

#### Natural products retarded DKD by regulating autophagy

Autophagy is an essential and evolutionarily conserved cellular mechanism responsible for breakdown and recycling of misfolded or malfunctioning proteins as well as damaged organelles to maintain cellular balance through lysosomal pathway [183–185]. Impairment of autophagy leads to injury in glomerular and tubular cells in experimental models of DKD, playing an intrinsic role in the development of disease [186–188]. Consequently, enhancing autophagic activity has emerged as a potentially effective therapeutic strategy to halt advancement of DKD [189].

Yiqi Jiedu Huayu decoction (YJHD), an adapted traditional Chinese remedy, has gained traction in treating DKD [190]. Research has shown that YJHD boosts levels of autophagy-related proteins LC3II and Beclin-1, while curbing p62, a testament to its autophagy-promoting potential. In addition, YJHD triggered AMPK pathway while dampening PI3K/Akt and mTOR pathways, suggesting its pivotal role in fostering autophagy [190]. Tangshen formula (TSF), a time-honored Chinese concoction of herbs, boasts potent healing powers for DKD [191]. This formula has been shown to mitigate podocyte apoptosis and damage, lower albuminuria levels, and improve kidney function in KKAy mice. TSF enhances nuclear translocation of transcription factor EB in diabetic podocytes, thereby boosting transcription of several autophagy-related genes [191]. The pharmacological activation of transcription factor EB by TSF expedited transformation of autophagosomes into autolysosomes and stimulated lysosomal development, thereby enhancing autophagic activity [191].

*Physalis peruviana* L. fruit extract, specifically ethyl acetate part, bolstered autophagic indicators, including LC3B and AMPK, while suppressing mTOR levels [192]. For millennia, Korean red ginseng has been a staple in traditional medicine for managing diabetes mellitus [193]. Korean red ginseng treatment led to an increase in autophagy marker LC3 and decrease in p62 levels. Concurrently, proteins pertinent to autophagy process, such as ATG7, are increased [193].

Several studies have suggested that many natural products ameliorate DKD by regulating autophagy (Tables 2 and 3). A recent study demonstrated that APS enhanced autophagy and attenuated DKD by targeting lncRNA Gm41268/PRLR pathway [115] (Table 1). Sarsasapogenin is the primary active component of *Anemarrhena asphodeloides* Bunge [194]. Sarsasapogenin, along with insulin, prevented decline in autophagy-related proteins such as autophagy-related 5, Beclin1, and light chain (LC) 3 B within kidney of diabetic rats [194]. Asiatic acid is a triterpenic acid sourced from *Cyclocarya paliurus* [195]. Asiatic acid can significantly alter levels of LC3 and lysosomal-associated membrane protein 1 proteins in TGF-β1-stimulated HK-2 cells treated by HG, implicating a possible mechanism of autophagy-lysosomal pathway in DKD fibrosis [195]. Quercetin enhanced LC3II/LC3I expression while decreasing p62 levels, suggesting that quercetin activates autophagy [196]. Experimental findings revealed that 2-dodecyl-6-methoxycyclohexa-2,5-diene−1,4-dione (DMDD), derived from *Averrhoa carambola* L., effectively lowered blood glucose levels and improved renal pathological changes in DKD mice [197]. In addition, in HG-induced HK-2 cells, DMDD attenuates excessive expression of glucose-regulated protein 78 (GRP78), C/EBP homologous protein (CHOP), LC3II/I, Beclin1, and autophagy related 5 [197]. Ferulic acid is a common phenolic acid found in plants and has antioxidant and anti-inflammatory properties [198]. Ferulic acid enhances autophagy by significantly promoting LC3 while inhibiting levels of p62 in kidney, thereby attenuating renal injury in diabetic mice [198]. *Cordyceps militaris* polysaccharides (CMP) supplementation effectively counteracted autophagy deficit induced by STZ, leading to a marked enhancement in renal autophagy rates [199]. Additionally, it stimulates levels of key autophagy-related proteins, including autophagy-related 5, beclin1, and LC3, while dampening expression of p62 [199].

#### Natural products abolished DKD by regulating AMPK signaling pathway

AMPK, a central regulator of cellular energy homeostasis, modulates mitochondrial biogenesis [200–203]. Previous research has demonstrated that diminished AMPK activation impairs mitochondrial biogenesis, leading to podocyte dysfunction and emergence of albuminuria in diabetic individuals [204, 205]. Moreover, AMPK has been shown to reverse autophagy and lower levels of AMPK are linked to root causes of DKD [206]. BaoShenTongLuo formula has been shown to mitigates damage in db/db mice and podocytes treated with HG by directly promoting mitochondrial biosynthesis through activation of AMPK and management of mitochondrial fusion processes [207]. Several studies have shown that natural products retard DKD by regulating AMPK signaling pathway (Tables 2 and 3). Geniposide, extracted from *Gardenia jasminoides* Ellis, is a potent iridoid glycoside [208]. Geniposide modulates AMPK pathways, thereby enhancing renal function and slowing development of DKD [208]*.* Kaempferol is a flavanol found in tea and various green vegetables [209]. Sheng et al. demonstrated that kaempferol attenuates apoptosis and enhances podocyte autophagy via AMPK/mTOR-mediated autophagic pathway in DKD mice [209]. Corilagin, a type of ellagitannin belonging to tannin family, is present in various medicinal plants [210]. Lou et al. showed that HG treatment decreases protein level of p-AMPK in podocytes, whereas treatment with sanguin reverses this change. These findings suggest that corilagin protects podocytes from HG-induced damage by augmenting expression of AMPK proteins [211]. Marein was derived from *Coreopsis tinctoria* Nutt. and is recognized for its ability to enhance DKD [212]. In HK-2 cells exposed to HG, Marein increased phosphorylated AMPK levels, which attenuated HG-induced cellular damage [212].

#### Natural products blunted DKD by inhibiting PI3K/AKT/mTOR signaling pathway

PI3K/AKT/mTOR signaling pathway is crucial for a variety of cellular functions [213–215]. It regulates essential processes, such as glucose balance, lipid metabolism, autophagy, apoptosis, and inflammation, significantly accelerating renal decline in DKD [216]. Further investigation showed that quercetin stopped HG-induced HK-2 cell apoptosis via PI3K/AKT signaling cascade [217].

Colquhounia root tablet hails from roots of *Tripterygium hypoglaucum* (H. Lév.) Hutch [218]. A previous study demonstrated that this drug reduced renal lipid deposition, inhibited inflammation, and curbed ECM protein synthesis in high-fat diet and STZ-induced DKD rat models. This mechanism operates by blocking activation of PI3K and AKT, consistent with findings from in vitro studies [219]. Network pharmacology analysis pinpointed 208 bioactive compounds within Jiedu Tongluo Baoshen formula, with PI3K/Akt/mTOR signaling pathway emerging as a promising candidate [220]. In kidneys of DKD rats, it suppresses expression of proteins linked to PI3K/Akt/mTOR pathway [220].

Previous studies have shown that natural products retard DKD by regulating PI3K/AKT/mTOR signaling pathway (Tables 2 and 3). Salvianolic acid B and tanshinone IIA work together to improve early DKD by increasing levels of phosphorylated forms of PI3K and Akt [216]. Isoorientin has been shown to robustly enhance autophagy in podocytes, offering substantial protection against damage caused by HG levels [221]. Using a proteomics approach, Isoorientin enhanced autophagy via suppression of PI3K-AKT-TSC2-mTOR pathway [221] (Fig. 2). Rutin, a polyphenolic flavonoid, is prevalent in various medicinal plants including *Sophora japonica*, *Astragalus membranaceus*, and *Buckwheat flowers* [222]. Reactivation of autophagy by rutin through activation of PI3K/AKT/mTOR signaling pathway attenuates endothelial-to-mesenchymal transition in db/db mice and HG-induced human renal glomerular endothelial cells [222]. Paeoniflorin is a vital component of herb *Paeonia lactiflora* Pall, which belongs to Ranunculaceae family [223]. Paeoniflorin contributes to restoration of STZ-induced autophagy in mouse podocytes and prevents cell death by utilizing PI3K/AKT signaling [223]. The findings indicated that solid-state fermentation products of *Radix astragali* and *Paecilomyces cicadidae* (RPF) exhibited a notable mitigating effect in DKD mice [224]. RPF has been shown to significantly lower levels of serum creatinine and urea in DKD mice. Morphological studies revealed that RPF not only improves structural integrity of kidney in DKD but also reduces podocyte apoptosis [224]. Additionally, in vitro experiments demonstrated that RPF boosts autophagy and safeguards podocytes by suppressing PI3K/AKT/mTOR signaling pathway [224].

#### Natural products dampened DKD by suppressing ER stress

Recent research has implicated ER stress as a pivotal driver of DKD pathogenesis [225, 226]. Elevated levels of ER stress markers, particularly GRP78 and CHOP, have been observed in kidney tissues of patients with DKD [227]. These molecular changes are frequently linked to structural problems such as glomerulosclerosis, tubular atrophy, and interstitial fibrosis [227]. Total glucosides of paeony, a potent extract from *Paeonia alba* root, packs a punch with anti-inflammatory, anti-apoptotic, antioxidant, and immunomodulatory effects [228]. Total glucosides of paeony decreased urinary protein levels in diabetic rats by suppressing ER stress-related markers [228]. Several studies have revealed that natural products retard DKD by regulating ER stress (Tables 2 and 3). Chrysin, a natural flavonoid, is mainly sourced from propolis, *Scutellaria baicalensis*, and *Oroxylum indicum* powers [229]. Chrysin mitigates ER stress by blocking PERK-eIF2α-ATF4-CHOP pathway, thereby helping to treat podocyte damage caused by excessive glucose levels and preserving slit diaphragm proteins [229]. Total flavones of *Abelmoschus manihot* (TFA) is a flavonoid isolated from *Abelmoschus manihot* flowers, and is a rich source of flavonoids [230]. TFA suppressed kidney inflammation and glomerular damage in DKD rats by alleviating ER stress [230]. Tan IIA, a compound sourced from *Salvia miltiorrhiza*, has a range of medicinal properties [231]. Tan IIA alleviates ER stress by inhibiting protein kinase RNA-like ER kinase (PERK) pathway, which ultimately reduces blood glucose, protects kidneys, and prevents fibrosis in STZ-induced DKD rats [232]. Arctigenin, a lignan isolated from *Fructus arctii*, reduced blood sugar and urinary protein levels in db/db mice, reducing kidney damage. In molecular biology, arctigenin fought ER stress by reducing GRP78, CHOP, and caspase−12 proteins, which subsequently mitigated cell death caused by glucose in HK-2 cells [233].

#### Natural products mitigated DKD by regulating JAK/STAT signaling pathway

JAK/STAT is the primary signaling node in the development and progression of DKD [234]. Derived from an ancient Chinese herb, wogonin has been shown to affect JAK/STAT pathway [235]. Over the last ten years, suppressor of cytokine signaling 3 (SOCS3) has been recognized as a key player in dampening inflammation, thereby negatively controlling inflammation associated with JAK/STAT pathway, a pivotal factor in the onset of insulin resistance and diabetes [235]. Several studies have shown that some natural products retard DKD by regulating JAK/STAT signaling pathway (Table 3). Previous studies have confirmed that wogonin markedly enhances SOCS3 levels, counteracting reduction in SOCS3 caused by hyperglycemia. Additionally, upregulation of SOCS3 levels triggered by wogonin notably decreased TLR4 levels, halted JAK/STAT pathway, and mitigated hyperglycemia-induced inflammatory response and activation of renin-angiotensin system [235]. This suggests that wogonin prevents renal cytotoxicity by promoting SOCS3 and inhibiting TLR4/JAK/STAT pathway [235].

#### Natural products abrogated DKD by regulating MAPK signaling pathway

Recent findings from both experimental and clinical studies indicate that p38-MAPK pathway is pivotal in the onset and progression of DKD [236]. Apigenin inhibited activation of downstream inflammatory MAPK pathway in STZ-induced diabetic rats [237]. Several studies have shown that natural products retard DKD by regulating MAPK signaling pathway (Table 3). Morin is a compound extracted from bark of Moraceae plants and various traditional Chinese herbs [238, 239]. Ke et al. found that morin inhibited growth of GMCs and prevented aggregation of fibronectin caused by HG levels, primarily by blocking p38 MAPK signaling pathway [240]. Baicalin, a prominent flavonoid isolated from medicinal plant *Scutellaria baicalensis* Georgi, has demonstrated numerous pharmacological effects, including antidiabetic, antioxidant, anti-inflammatory, and anticancer effects [241]. By suppressing MAPK-mediated inflammatory response, baicalin was able to diminish production of pro-inflammatory cytokines such as interleukin-1β and interleukin-6, thereby alleviating inflammatory damage in DKD models [241]. Hirudin, a key component extracted from leech salivary glands, is a tiny protein made up of approximately 65–66 amino acids [232]. In addition, Han et al. confirmed that hirudin could restrain inflammation and inhibit podocyte apoptosis through p38 MAPK/NF-κB pathway in a rat model of DKD, protecting kidney from injury [232]. G-Rg3 is a unique saponin extracted from either red or black ginseng, and is created by treating original saponin with acid at high temperatures, which causes sugars to isomerize [242]. This compound has two forms, known as 20(R)-Rg3 and 20(S)-Rg3, and they differ based on placement of hydroxyl group on carbon-20, each serving distinct roles [242]. MAPK phosphorylation markers such as phosphorylation of extracellular signal-regulated kinase, JNK, and p38 were significantly elevated in DKD mice, but were significantly reduced by 20(R)-Rg3 treatment, suggesting that MAPK pathway is inhibited and inflammatory stress is reduced in DKD mice [242].

## Conclusion, challenges, limitations, and perspectives

Natural products, including TCM, are considered as a unique medical system. The multi-component characteristic of TCM allows for hitting multiple targets through various components. It embodies holistic therapeutic effects; the form of combination drug formulas enhances efficacy and reduces drug side effects and drug resistance through multi-component therapy. Therefore, the study of TCM treatment on various diseases has attracted widespread attention, particularly its unique advantages in treating complex, multifactorial diseases such as metabolic diseases, including DKD. Although significant progress has been made in the isolation and identification of compounds in botanicals or herbs, bottlenecks and challenges remain in the methodological development of revealing the overall efficacy and synergistic effects of multi-component drugs, especially TCM formulas. Emerging multiomics, by integrating multi-dimensional data from genome, transcriptomics, proteomics, and metabolomics, can systematically construct a complete causal chain from genetic blueprint to immediate physiological phenotype, thereby revealing body true state at the molecular level. This promotes high-throughput screening and clinical diagnosis in the pharmaceutical industry and provides a new perspective for evaluating overall efficacy of TCM.

The beneficial effects of natural products on DKD are closely linked to pharmacological effects, such as metabolic regulation, antioxidant activity, anti-inflammatory responses, antifibrotic properties, and protective effects on renal tubules and glomerular damage. This review presents that natural products offer a promising multi-target therapeutic strategy for the treatment of DKD by regulating AGE/RAGE, IκB/NF-κB, Keap1/Nrf2, TGF-β/Smad and Wnt/β-catenin signaling pathways that are associated with multiomics-related mechanisms, including GM dysbiosis, aberrant ncRNA transcription, abnormal proteome expression, and metabolite disorder. Increasing publications have suggested that targeting metagenome, transcriptome, proteome and metabolome are a promising therapy for the treatment of DKD. The multiomics studies highlight that the pharmacological effects are associated with aberrant regulation of metagenome, transcriptome, proteome and metabolome. Therefore, the underlying molecular mechanism by which natural products ameliorate DKD by reshaping GM dysbiosis, restoring aberrant expression of ncRNAs and regulating metabolite disorder will provide new therapeutic targets for treatment of DKD of natural products.

Although multi-omics approaches can clarify intervention process of natural products in DKD, evaluating omics data in isolation does not always prove causality between omics findings and downstream molecular signals, such as AGE/RAGE, IκB/NF-κB, Keap1/Nrf2, TGF-β/Smad and Wnt/β-catenin pathways. Furthermore, caution must be taken to limit potential erroneous findings from integrating large datasets. Although appropriate statistical methods play a crucial role in identifying and correcting erroneous findings, integrating multiomics datasets with in vivo and in vitro experimental models can demonstrate the causal relationship between natural products and DKD intervention. Moreover, the transcriptional profiles of genes and cell lines during DKD development can be addressed by comparing genome-wide association studies. This multiomics approach is beginning to address one of the major shortcomings of the field.

Previous publications have clarified the upstream molecular mechanisms by which natural products inhibiting excessive accumulation and deposition of ECM components by antioxidant, anti-inflammatory, and anti-fibrotic mechanisms in DKD. However, previous publications focus on underlying molecular mechanistic exploration and animal model validation, facing significant hurdles in translating these discoveries into clinical practice. Several critical challenges, such as compositional complexity, quality control, herb-drug interactions, dose–response relationships, pharmacokinetics, low bioavailability, and long-term safety remain important barrier limiting clinical application of natural products. Moreover, the exploration of natural products for treating DKD is in its early stages, ongoing and thorough investigations into these compounds and their mechanisms give us reason to be optimistic about future advancements in this area, ultimately enhancing treatment options for DKD. Therefore, future studies should focus on establishing the map of an integrated multiomics-mediated signaling pathway and developing precision stratification strategies according to biomarkers to promote personalized therapies targeting multiomics-mediated signaling network. Furthermore, future research should strive to combine basic research with clinical validation to achieve the translation of basic research into clinical practice. This can overcome the limitations of current strategies through well-designed clinical trials, optimized drug delivery systems, and the elucidation of bioactive components.

## Data Availability

No datasets were generated or analysed during the current study.

## Abbreviations

AGE: Advanced glycation end products
AKT: Protein kinase B
AMPK: Adenosine 5'-monophosphate-activated protein kinase
APS: Astragalus polysaccharides
AS-IV: Astragaloside IV
BHID: Bekhogainsam decoction
CAPE: Caffeic acid phenethyl ester
CKD: Chronic kidney disease
DKD: Diabetic kidney disease
DMDD: 2-Dodecyl-6-methoxycyclohexa-2,5-diene−1,4-dione
ECM: Extracellular matrix
ER: Endoplasmic reticulum
ESRD: End-stage renal disease
GBM: Glomerular basement membrane
GDC: Gandhi capsules
GM: Gut microbiota
GMCs: Glomerular mesangial cells
HG: High glucose
HKC: Huangkui capsules
HRMCs: Human renal mesangial cells
IPA: Indole−3-propionic acid
IκB: Inhibitor of kappa B
JAK: Janus kinase
Keap1: Kelch-like ECH-associated protein 1
lncRNA: Long non-coding RNA
LPS: Lipopolysaccharide
MLB: Magnesium lithospermate B
mTOR: Mammalian target of rapamycin
ncRNAs: Non-coding RNAs
NF-κB: Nuclear factor kappa B
Nrf2: Nuclear factor erythroid 2-related factor 2
PERK: Protein kinase RNA-like ER kinase
PI3K: Phosphoinositide 3-kinases
PKC: Protein kinase C
PRLR: Prolactin receptor
QYDP: Qishen Yiqi dripping pill
RAGE: Receptor for advanced glycation end products
RAP1: Ras-proximate−1
SBL: Sea buckthorn leaves
SHYS: San-Huang-Yi-Shen capsule
Smad: Suppressor of Mothers against decapentaplegic
SOCS3: Suppressor of cytokine signaling 3
STAT: Signal transducers and activators of transcription
STZ: Streptozotocin
TA: Thonningianin A
TCM: Traditional Chinese medicines
TGF-β: Transforming growth factor β
TSF: Tangshen formula
UACR: Urinary albumin-to-creatinine ratio
VEGF-A: Vascular endothelial growth factor A
YJHD: Yiqi Jiedu Huayu decoction
